# Development of a novel electroporation method for the oyster parasite *Perkinsus marinus*

**DOI:** 10.1038/s41598-022-24548-0

**Published:** 2022-11-21

**Authors:** Hirokazu Sakamoto, Xiaoxia X. Lin, Yun D. Bai, Xue F. Chen, Ziyue Z. Zhang, Yui Honjo, Kenji Hikosaka

**Affiliations:** 1 Department of Infection and Host Defense, Graduate School of Medicine, Chiba University, Chiba, Japan; 2 Department of Biomedical Chemistry, Graduate School of Medicine, The University of Tokyo, Tokyo, Japan

**Keywords:** Parasite genetics, Transfection

## Abstract

Gene manipulation techniques are fundamental to molecular biology and are continuously being improved. However, gene transfection methods are not established for many unicellular eukaryotes (protists), thereby hindering molecular biological investigations. The oyster parasite *Perkinisus marinus* is one of the few protists with established gene transfection and drug selection. Nevertheless, the present protocols are tedious, requiring a specific electroporator and pulse conditions which limits the accessibility of this technique across different research groups. Here, we present alternative buffer and electroporation conditions that make the protocol less restrictive. We revealed the pulse condition that enables the introduction of plasmids into *P. marinus* cell using Ingenio electroporation buffer and NEPA21 electroporator. We found that number of cells and plasmid concentration were critical parameters for the electroporation system. We also constructed a simpler expression plasmid that is removed needless regions for gene expression in the parasite. Our findings resolved the equipment restriction in electroporation of *P. marinus* and would be a good reference for electroporation in other protists, in particular other Perkinsozoa parasites and core dinoflagellates.

## Introduction

**Figure 1:**
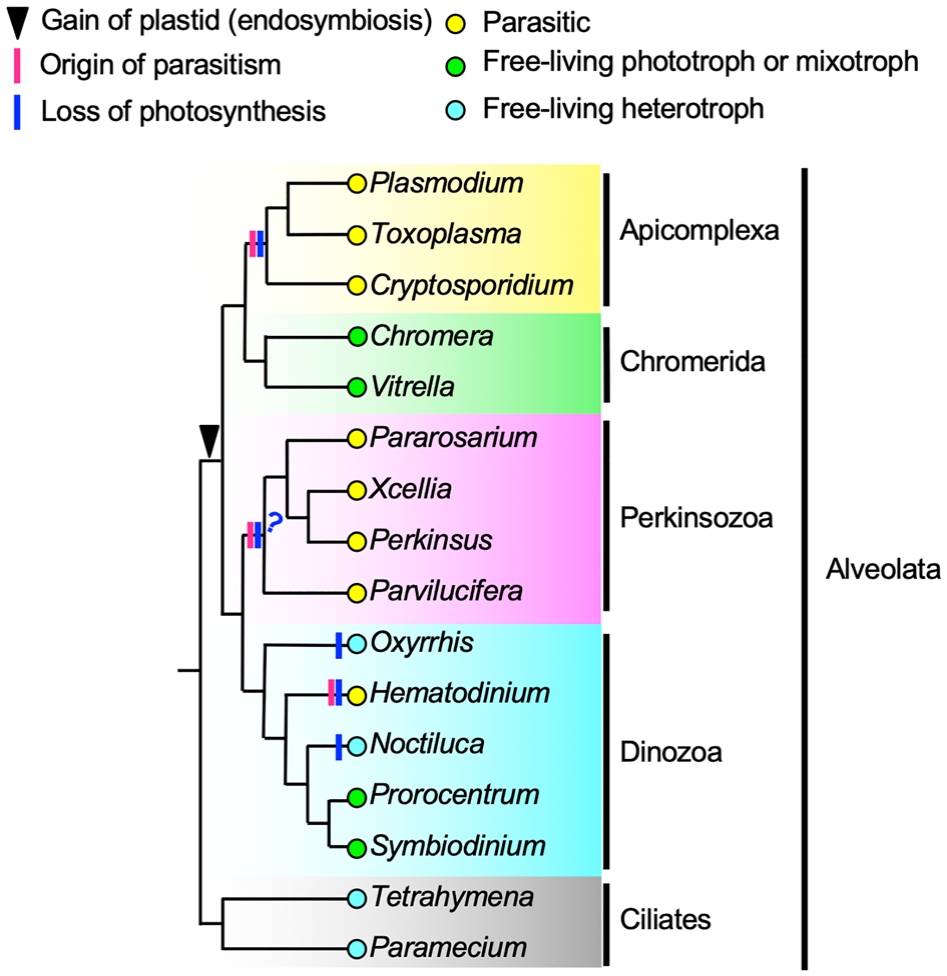
Simplified phylogenic tree of the Alveolata and the evolutionary background of its complicated lifestyle. Traditionally, Alveolata was divided into three main groups: ciliates, Dinozoa, and Apicomplexa. Acquisition of photosynthesis (the black triangle) by secondary endosymbiosis occurred in the common ancestor of Dinozoa, Perkinsozoa, Chromerida, and Apicomplexa. However, loss of photosynthetic capacity (shown by the blue bars on the tree) and acquisition of parasitism (shown by the magenta bars on the tree) independently occurred multiple times. Thus, there is a complicated mixture of free-living and parasitic protists in Alveolata. Cellular and molecular biological studies are intensive in *Plasmodium* and *Toxoplasma*, for which gene transfection methods have been established. *Perkinsus* is also expected to become a new model organism closely related to Apicomplexa, as progress is being made in gene transfection technology. The question mark at the base of Perkinsozoa indicates uncertainty about the timing of the loss of photosynthesis.

*Perkinsus* is a unicellular marine parasite that is a member of Perkinsozoa^1,2^ (Fig. 1). *Perkinsus marinus* causes Dermo disease in the wild and cultivated eastern oyster, *Crassostrea virginica*, and has caused significant economic losses^3,4^. Since there is no way to prevent the spread of *Perkinsus* infection, the parasite is listed in the World Organization for Animal Health (OIE)^2^. For effective countermeasures, basic biological studies are required.

Beyond the importance of fisheries, *Perkinsus* spp. are uniquely important in evolutionary biology owing to their placement in the eukaryotic tree of life (Fig. 1). *Perkinsus* spp. is nested within the Perkinsozoa that is branched from the base of the Dinozoa. Perkinsozoa includes genera of *Perkinsus*, *Xcellia*, *Pararosarium*, and *Parvilucifera* etc.^2,5–11^. They are all parasitic and thus acquired parasitism in their common ancestor. This acquisition of parasitism evolved independently of the closely related Apicomplexa. The common ancestor of Myzozoa (Dinozoa, Perkinsozoa, Chromerida, and Apicomplexa) was a phototroph^12,13^; previous results have shown that photosynthesis has been lost independently multiple times^13,14^. Therefore, molecular biological analyses between Perkinsozoa and Apicomplexa will be necessary for understanding the parasites’ pathogenesis and also the principle of the degeneracy of genome and cellular functions due to parasitism. Because comparative analysis with Apicomplexa provides a generalization of the evolution caused by parasitism, research using *Perkinsus* is attracting increasing attention from researchers in various fields^12,15–18^.

Gene manipulation techniques are fundamental to molecular biology and are continuously being improved. However, transgenic methods are still lacking for many non-model organisms, hindering our understanding of their unique cellular systems, although several important progresses have been achieved in the gene transfection project for non-model marine protists^19^. *Perkinsus marinus* is a rare protist that has established an electroporation method^20^ and also drug selection systems^16,21,22^. Thus, *P. marinus* is an influential model for elucidating the unique biology of its relatives, in particular Dinozoa and other Perkinsozoa parasites. However, only the specific equipment for the electroporation has been reported^20^. Furthermore, the electric pulse conditions are not open, making it difficult to develop electroporation methods using other equipment. In other words, the most critical problem is that there is only one option for the electroporation of *P. marinus*: specific equipment and a specific buffer. Furthermore, it is expensive. For molecular and cellular biological research in *P. marinus* to spread to more researchers and promote the above research fields, it is essential that flexibility be ensured in the equipment and buffers for gene transfection methods. Therefore, in this study we have developed a new electroporation system that is independent of the existing method.

To overcome the limits, we screened electroporation buffers using Amaxa Nucleofector II, the only device currently used for *P. marinus* transfection and identified a new alternative buffer. Then we examined pulse conditions using the buffer by NEPA21 electroporator, which can freely control the voltage and the frequency and intervals of the pulses. For the first time, we clarified the pulse conditions for *P. marinus*. Our findings will significantly expand the possibilities for transfection using more other equipment. Moreover, we newly designed simpler plasmids suitable for gene expression in the parasite, which enables gene manipulation experiments more readily and rapidly. The information will contribute to the molecular-biological studies of *P. marinus*.

## Results

### Identification of a new buffer working in the conventional condition

To date, only the D-023 program of the Amaxa Nucleofector is used for transfection of *Perkinsus marinu*s^20^. There are no reported successes with other equipment. We first screened buffers (Table 1) compatible with the D-023 program in the Amaxa Nucleofector using 50 × 10^6^ cells (CB5D4 strain, a strain used in the first report of transfection^20^) to develop a new electroporation method using 10 µg of pMGB plasmid^23^. Transfection efficiency was evaluated on a 5-point scale using GFP-positive cells with the original buffer, Solution V in a Transfection Kit (Lonza), being 100%. Cytomix^24^ and 3R buffer^16^ without CaCl_2_ did not work, while 3R buffer with CaCl_2_ worked as previously reported^16,25^ (Table 1). Although the transfection efficiency was low, we found that the Ingenio electroporation solution also worked in the D-023 program (Table 1).

**Table 1:** Comparison of electroporation efficiency using the D-023 program in Amaxa Nucleofector II among electroporation buffers.

Solution	Fluorescence level
Solution V (Lonza)	+ + + + +
Cytomix	−
3R	−
3R + CaCl_2_	+ + + +
Ingenio (Mirus)	+ +

### Estimation of the voltage conditions for possible gene transfer

To estimate the voltage condition in the D-023 program, we used another electroporator, NEPA21, to refine the voltage conditions. This NEPA21 generates square electric pulses and can freely control the voltage, and the frequency and intervals of the pulses, and has been successfully transfected into the green alga *Chlamydomonas reinhardtii* and diatom *Phaeodactylum tricornutum*^26,27^. We were able to transfect the CB5D4 strain (50 × 10^6^ cells) with 10 µg of the pMGB plasmid using the conventional Solution V at 100–200 V, albeit with low efficiency using the NEPA21 (Table 2). This is the first report that breaks through the limitations of the electroporation equipment and provides a critical research motivation to examine conditions with equipment other than Amaxa Nucleofector.

**Table 2:** Comparison of electroporation efficiency using the Lonza buffer and NEPA21 electroporator.

Solution	Pulse	Fluorescence level
Solution V (Lonza)	100 V × 2	+
Solution V (Lonza)	100 V × 6	+ +
Solution V (Lonza)	200 V × 2	+

**Figure 2:**
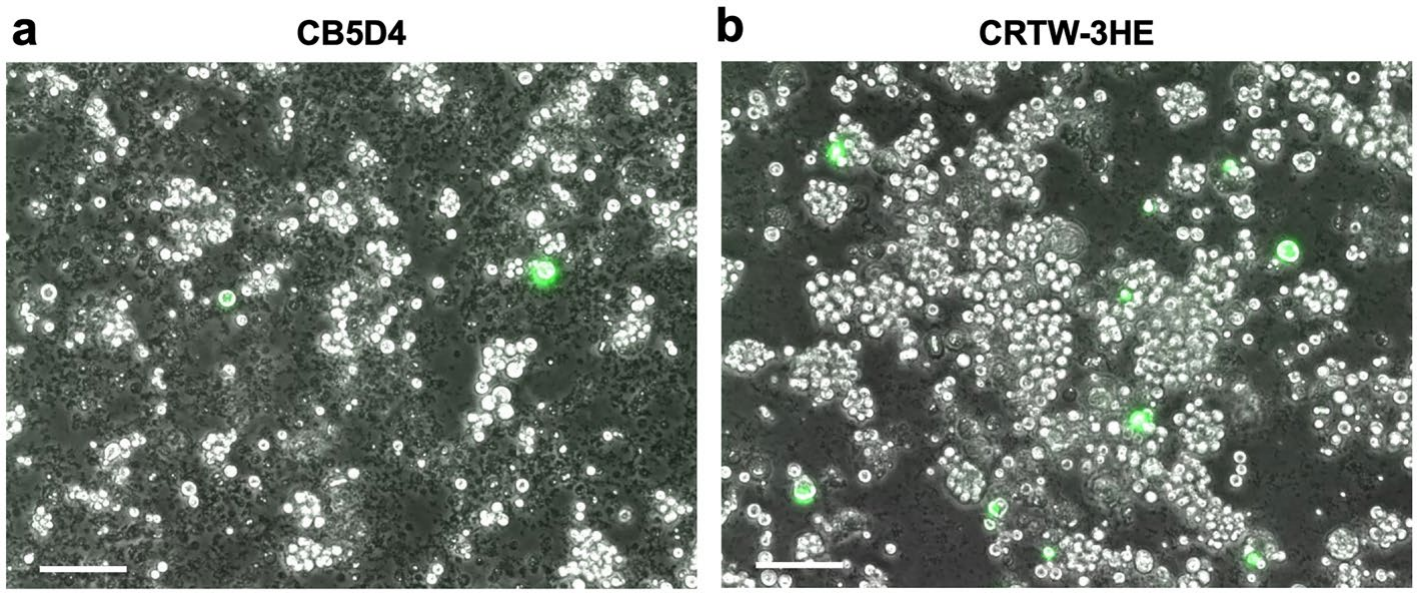
Differences in cell damage between cell strains. 10 × 10^6^ cells were electroporated with 5 µg of the plasmid by 175V × 5 pulses. Images were taken three days after the transfection. Merge images of GFP and bright field are shown. The white and spherical objects observed indicate living cells. Other objects observed as small, black shadows indicate cellular debris. Many cell debris can be observed in the CB5D4 strain (**a**), whereas very few were observed in the CRTW-3HE strain (**b**). The scale bars; 50 µm.

Next, cell amount and voltage were screened to establish the new electroporation method using the Ingenio buffer (Table 3). In this buffer, overcurrent error (E01 error) frequently occurred when the plasmid amount was 10 µg, so we tested with 5 µg. We found the following two things; (1) 175 V × 5 pulses is much better than 175 V × 2 pulses at 50 × 10^6^ cells, (2) 200 V × 2 pulses is much better than 200 V × 1 pulse at 10 × 10^6^ cells. The results are the first case of high-efficiency transfection using a device and a buffer other than Lonza products (Table 2 and Fig. 2a).

**Table 3:** Comparison of electroporation efficiency using the Ingenio buffer and NEPA21 electroporator.

Cell# (× 10^6^)	Pulse	Fluorescence level	Cell debris level
50	175 V × 2	+	N.D
50	175 V × 5	+ + + +	N.D
50	200 V × 1	+ + +	N.D
50	200 V × 2	E01 error*	N.D
10	125 V × 5	+	N.D
10	150 V × 5	+ +	+ +
10	175 V × 5	+ +	+ +
10	200 V × 1	+	+ + + +
10	200 V × 2	+ + + +	+ + + +
10	200 V × 5	+ + +	+ + + +

N.D.: Not detected.

*Overcurrent error.

However, the overcurrent error occurred 20–30% at 50 × 10^6^ cells. Thus, we reduced the cell number to 10 × 10^6^ cells; no errors occurred, and high transfection efficiency with 200 V × 2 pulses (Table 3). However, much cell debris was observed at 10 × 10^6^ cells (Fig. 2a).

### CRTW-3HE strain is more suitable for electroporation than the CB5D4 strain

Following electroporation at 10 × 10^6^ cells in the Ingenio buffer using the NEPA21 electroporator, we observed a lot of cell debris of CB5D4 cells (Fig. 2a), suggesting the pulse killed many cells. However, the same conditions did not produce the same observation when applied to *P. marinus* strain, CRTW-3HE (ATCC50439), a strain used in development of the drug selection system^21,22^, with similar transfection efficiency (Fig. 2b). The finding indicates that the CRTW-3HE strain is more suitable for transfection experiments than CB5D4 strain. That is why we use the CRTW-3HE strain in the following experiments.

### Determination of fine pulse condition

We optimized the pulse conditions of the NEPA21 electroporator using the CRTW-3HE strain. Since increasing cell number increases error frequency, we validated at 10 × 10^6^ cells and 5 × 10^6^ cells. The plasmid amount was 5 µg based on the previous study^20^, and 10 µg was also tested to assess whether the transfection efficiency is further increased. Pulse conditions were tested in the two conditions identified in Table 3, 175 V × 2 pulses and 200 V × 2 pulses.

**Figure 3:**
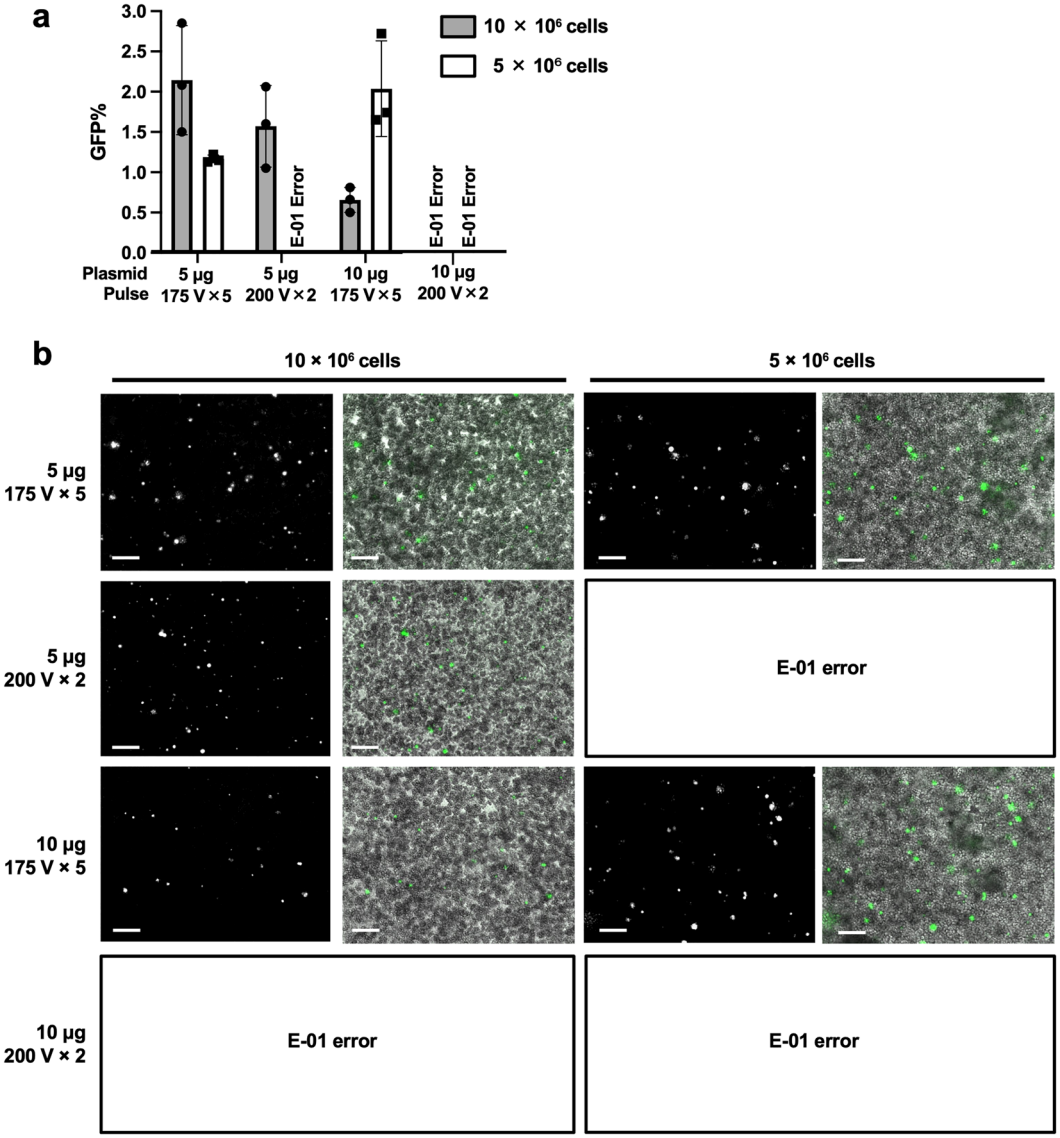
Comparison of GFP signals among the indicated pulse conditions and plasmid amount in the CRTW-3HE strain. (**a**) Percentage of GFP-positive cells after electroporation in each condition. The dots indicate individual data, the bars indicate mean values of the data, and the error bars indicate standard deviation. (**b**) GFP images (left side) and merge images (right side) are shown for each condition. The scale bars; 50 µm. E01-error in **a** and **b**; overcurrents.

We found that the 175 V × 5 pulses with 5 µg plasmid enables transfection with high efficiency, approximately 2% (Table 4 and Fig. 3a and b); this value is enough for the next experiment, drug selection. In the previous report^25^, the transfection efficiency was increased by reducing the cell number and increasing the plasmid amount; the plasmid/cell ratio was tenfold higher from the other report^20^ (to 1.0 pg/cell from 0.1 pg/cell). Therefore, we tested electroporation with the plasmid/cell ratio was 1.0–2.0 pg/cell. However, there was no significant improvement in efficiency. Finally, we determined the optimal pulse condition to be 10 × 10^6^ cells, 5 µg of plasmid, and 175 V × 5 times. We used this condition in the following experiments.

**Table 4:** Fining of pulse condition using the Ingenio buffer and NEPA21 electroporator.

Cell# (× 10^6^)	Plasmid (µg)	Plasmid/cell (pg/cell)	Pulse	GFP rate (%)
10	5	0.5	175 V × 5	2.14
			200 V × 2	1.57
10	10	1	175 V × 5	0.66
			200 V × 2	E01 error*
5	5	1	175 V × 5	1.17
			200 V × 2	E01 error*
5	10	2	175 V × 5	2.04
			200 V × 2	E01 error*

*: Overcurrent error.

### New expression plasmids for *P. marinus*

**Figure 4:**
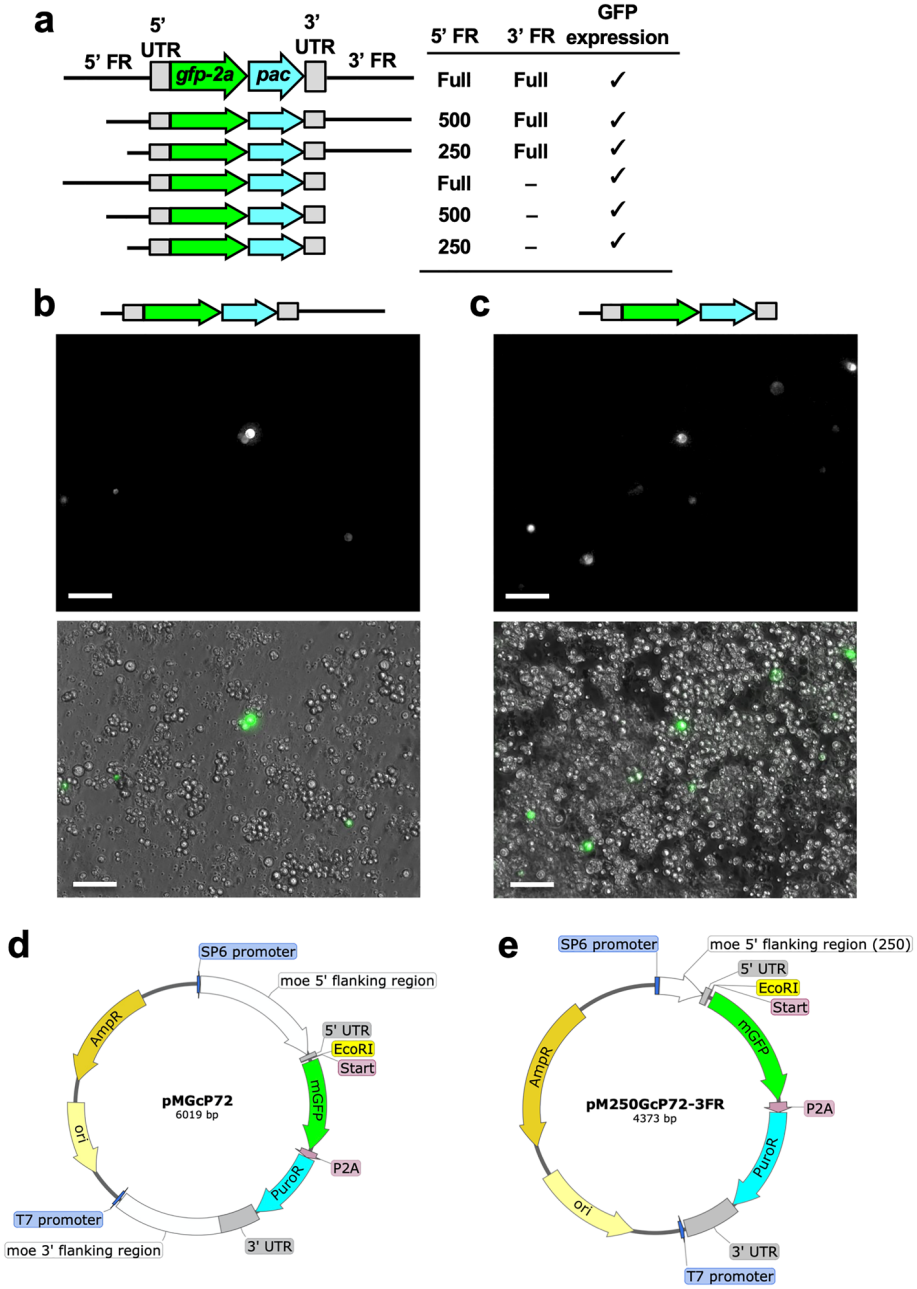
Requirement of 5' and 3' flanking regions of *moe* gene for GFP expression in the CRTW-3HE strain. (**a**) Schematic diagrams between 5′ and 3′ flanking regions (FR) of the tested plasmids (left side). The length of FRs and results of GFP expression are shown on the right side. (**b** and **c**) Representative images of GFP expression by promotor activity of 250 bp 5' FR with and without 3' FR are shown in (**d**) and (**e**), respectively. The scale bars; 50 µm. Schematic diagrams of pMG_C_P72 (**d**) and pM250GcP72-3FR (**e**) are shown. The images are provided by SnapGene software.

The expression plasmids for *P. marinus* were constructed based on pCR4-TOPO vector^20^. The plasmid has extra sequences not involved in gene expression in the parasite cell. Since the molecular mechanism is unknown in establishing stable expression cells in *P. marinus*, removing as many sequences as possible that are irrelevant to expression is desirable to avoid unforeseen events and establish stable cells reproducibly. Therefore, we constructed a new expression plasmid with a simpler sequence based on the pSP72 vector. The exclusion of the kanamycin resistance gene and the LacZa gene in the pCR4-TOPO resulted in approximately 1.6 kb shorter plasmid length. The plasmid contains the replication start site (ori), ampicillin resistance cassette, the *moe* gene promoter and the 5' and 3' UTR of the *moe* gene, and puromycin resistance gene (puromycin N-acetyl-transferase: pac). We named the plasmid pMG_C_P72 (pMOE-GFP-C terminal-Pac in pSP72) (Fig. 4d). Electroporation of the plasmid using NEPA21 results in the expression of GFP (Fig. 4b). This demonstrated that the simplified plasmid with different backbone does not affect gene expression in *P. marinus*.

To further exclude needless sequences, the necessity of 5′ and 3′ flanking regions was tested. For the 5' flanking region, even the shortest 250 bp region had promoter activity (Fig. 4a,b, and c). Moreover, for the 3' flanking region, exclusion of this region did not affect gene expression (Fig. 4a and c). Taken together, most of the flanking region sequences of the conventional plasmid are unnecessary for gene expression. Thus, we designed a new plasmid with minimum flanking regions (FR). We named the plasmid pM250G_C_P72-3FR (pMOE 250-GFP-C terminal-Pac without 3’ FR in pSP72) (Fig. 4e).

## Discussion

Because of recent reports of the development of genetic engineering techniques, *Perkinsus marinus* is becoming important as a model organism for biology associated with parasitism^15,16,23,25^. However, the high cost of the electroporation experiments is the problem. Recently, a new transfection buffer, 3R buffer, has made it possible to perform electroporation inexpensively for the parasite^16,25^, but with the limitation that the specific equipment, Amaxa Nucleofector, must be used. In this study, we succeeded in determining for the first time the pulse conditions under which transfection is possible using different equipment, the NEPA21 electroporator. Equipment limitation in electroporation for *P. marinus* is significantly reduced by this study.

We first screened transfection buffers using the Amaxa Nucleofector and found that the Ingenio buffer yielded sufficient transformants. We further identified the pulse conditions using the NEPA21 electroporator. Finally, we determined the optimal pulse condition to be 10 × 10^6^ cells, 5 µg of plasmid, and at 175 V × 5 pulses. The GFP transfection efficiency was approximately 2% using this condition, comparable to the 3R buffer-based transfection in the previous report (2.52%)^16^, which is enough for drug selection. Furthermore, this condition gave adequate results with fewer cells and fewer plasmid amounts than a previous report^25^. Remarkably, in our trial, the ratio of the plasmid and cell number did not correlate with the transfection efficiency, and even the previous report^25^ optimized ratio (2 pg/cell) was not optimal in our system, the combination of the Ingenio buffer and the NEPA21 electroporator. The transfection efficacy should be not only determined by the ratio of the plasmid and cell number, but the characteristics of the electroporator and buffer are also considered important factor. Therefore, establishing transfection methods with new equipment, it will be required to examine various parameters in an unbiased manner.

We, for the first time, found that pulse damage differs between *P. marinus* cell strains, although only two strains were examined. Since differences in pulse-induced cell damage between cell strains have not been reported, the results provide critical information for the future development of genetic manipulation techniques for *P. marinus*. In the future, it may be necessary to screen cell strains that are more tolerant to pulses and have higher transfection efficiency. In order to establish *P. marinus* as a model organism, cell strains that are suitable for genetic manipulation and molecular biological experiments should be identified.

In this study, we also proposed a new simpler plasmid that appears more suitable for gene expression of *P. marinus* cells. It is unclear how foreign DNA sequences are integrated into the genome in *P. marinus*, but it has been suggested that they may be integrated by transposons^19^. Thus, nucleotide sequences unrelated to gene expression could be integrated with the target gene. This may result in the appearance of unexpected phenotypes other than the expression of the target gene. To minimize this risk, we constructed a plasmid that eliminated as much as possible of the sequences other than those necessary for gene expression and plasmid selection within the bacteria from the pMGP plasmid^23^. We demonstrated that the new simpler plasmid expressed the target gene in the parasite cell, and 5′ and 3′ flanking regions of the *moe* gene were not needed for its expression. Our results would be significant for future strategies to generate stable expression in *P. marinus* cells.

In conclusion, we determined for the first time the pulse conditions for *P. marinus* transfection using the NEPA21 electroporator. This condition will provide the basis for establishing transfection using more other equipment. Developing transgenic technology that is less expensive than conventional methods and not restricted by equipment would facilitate the widespread use of *P. marinus* research. Our findings would also provide critical information for establishing new electroporation methods in closely related species, such as other Perkinsozoa parasites and core dinoflagellates.

## Methods

### Parasite cell culture

*P. marinus* strains CRTW-3HE (ATCC 50439), and CB5D4 (PRA-240) were purchased from the American Type Culture Collection (ATCC). The cells were maintained at 26 °C in the ATCC medium 1886, and subcultured 0.1–1.0 ml of cells into 10 ml of fresh medium in a T-25 culture flask once every 1–3 weeks. For experiments, exponential growth phase cells were subcultured once every 3 days.

### Plasmid construction

To construct the pSP72 vector-based new plasmids, we used a pCR4-TOPO vector-based pMGP plasmid^23^ as a PCR template to obtain the following insertions. For pMG_C_P72 (pMOE-GFP-C terminal-Pac in pSP72) plasmid (Fig. 4d), from the 5′ to 3′ flanking regions (FR) of the *moe* gene, including monomeric GFP (*mgfp)* and the puromycin resistant gene (puromycin N-acetyl-transferase; *pac)* genes, was amplified using the primers moe5FR-full-F (5'-TAGGT GACAC TATAG AACTC GAGTC TCGTA ATGAG CCCAA CCATT AT-3') and moe3FR-full-R (5'-ACTAT AGGGA GACCG GCAGA TCTGG AGGAC TTGAG GCTCT GTGAC-3'). For pM250G_C_P72-3FR (pMOE 250-GFP-C terminal-Pac without 3′ FR in pSP72) plasmid (Fig. 4e), we use the primers moe5FR-250-F (5′-TAGGT GACAC TATAG AACTC GAGCC TTCAT TGTAT GCGTG AGTAT GT-3′) and moe3UTR-R (5'-ACTAT AGGGA GACCG GCAGA TCTCT GCACT CTCCC AACGC AACAC GA-3′). For amplification of 500 bp of the 5′ flanking region of the *moe* gene, the primer moe5FR-500-F (5'-TAGGT GACAC TATAG AACTC GAGTT GCTCC GGCTT GTAGT GAACT AAC-3') was used as a forward primer, and moe3FR-full-R or moe3UTR-R were used as reverse primers. The amplicons were ligated with the XhoI, and BglII cut pSP72 using Gibson Assembly Master Mix (New England Biolab, Ipswich, MA).

### Electroporation

Electroporation using an Amaxa Nucleofector II (Lonza, Basel, Switzerland) was performed as described in the previous study^21^. The other machine NEPA21 electroporator (Nepa Gene, Chiba, Japan) can control two types of pulses: poring and transfer. We determined the pulse conditions for *P. marinus* as follows; Voltage (V): 175, msec: 1, Pulse number: 5, Interval (msec): 50, Decay (%): 10, Polarity: + /− for poring pulse, and Voltage (V): 20, msec: 50, Pulse number: 10, Interval (msec): 50, Decay (%): 40, Polarity: + /− for transfer pulse. For the experiments in Table 2, we did not change the transfer pulse condition. 5 or 10 µg plasmid was ethanol-precipitated, and the pellet was resuspended in 100 µl of the Ingenio electroporation solution (Mirus Bio, Madison, WI). 5 × 10^6^ cells were collected by centrifugation at 1000 × *g* for 3 min at room temperature. The cell pellet was resuspended in the plasmid-containing Ingenio solution and transferred all into a 2-mm cuvette (Nepa Gene), and electroporated by NEPA21 electroporator using the pulses described above. The treated cells were transferred into a well in a 12-well plate with 2 ml of fresh ATCC medium 1886. Then, it was divided into 1 ml × 2 wells and incubated at 26 °C. The percentages of the GFP-positive cells were calculated by counting at least 3000 cells by 3 individuals 1 week after the electroporation.

### Fluorescence microscopy

Live cells were imaged using IX73 (Olympus, Tokyo, Japan), and acquired data were processed with ImageJ software (National Institutes of Health (NIH), Bethesda, MD).

## Data Availability

All data generated during this study are included in this article.
